# The Burden of Physicians’ Administrative Workload Affects Clinical and Scientific Mentoring

**DOI:** 10.15694/mep.2019.000074.1

**Published:** 2019-04-01

**Authors:** Anna Nia, Dmitry Zavlin

**Affiliations:** 1 University of Texas Medical Branch; 2 Drexel University College of Medicine

**Keywords:** administration, electronic health record, billing, faculty, students, residents, teaching

## Abstract

This article was migrated. The article was marked as recommended.

## Letter

Dear Sir,

Trust has always been considered as a fundamental attribute of medical profession that gives physicians the foundation to build their professional relationships on. Trust has also been critical in a patient’s compliance to treatment and adherence to follow-up appointments. In the same vein, young students on clinical rotations or medical graduates who begin their residency have to trust their physician mentors with their graduate and postgraduate training. Unfortunately, most trainees have heard from their faculty members that the increasing administrative burdens associated with their clinical and academic roles are often exhaustive and redundant ( Woolhandler and Himmelstein, 2014). A vast majority of institutions is operated by staff without medical education ( Jauhar, 2017). These trends typically originate from stricter insurance regulations on spending and complex compensation algorithms that require immense billing-related activities in our US multi-payer healthcare system ( Jiwani *et al.*, 2014). In addition, many physicians are part of different academic committees and have duties to maintain and acquire funding for scientific research.

This non-medical workload ultimately jeopardizes mentors’ ability to teach their trainees, and cultivate an intellectual environment to discuss the current literature and stay up-to-date with cutting edge medical technology. These increasing demands on faculty members are also one of many reasons for stagnating physician- and surgeon-scientists ( Schafer, 2010; Kibbe and Velazquez, 2017). Face-to-face time between mentees and mentors is still one of the most important requirements to develop a close relationship that would foster an intellectually stimulating environment resulting in mutual trust and consequently a quality education or scientific advancement ( Straus *et al.*, 2013). A lack of availability may diminish the trust in a mentoring relationship and lead to poor mentee progress.

Simplification and optimization processes in the economic and regulatory structures of US healthcare are necessary to open time and resources of faculty physicians and strengthen both the clinical education and the scientific advancement of young trainees. Automatization of recurring duties, delegation of basic tasks to non-doctoral staff, and the use of human assistance technology (i.e. dictation devices) are further measures that can be taken by attending physicians ( Rao *et al.*, 2017) as well as residents ( Podnos *et al.*, 2003).

## Notes On Contributors

Ms. Anna M. Nia graduated from UCLA with a B.S. in Neuroscience (2012) and a M.S. in Bioengineering (2015). She is currently a M.D.-Ph.D. student at the University of Texas Medical Branch conducting large data analysis using various bioinformatics and machine learning tools collected from different experimental conditions. Ms. Nia developed and drafted the manuscript.

https://orcid.org/0000-0003-2757-7267

Dr. Dmitry Zavlin graduated with distinction from the medical school of the Technical University of Munich (2015) and subsequently finished a 2-year postdoctoral fellowship at the Houston Methodist Hospital where he published numerous peer reviews articles. He currently works as a resident physician at a general surgery program in Easton, Pennsylvania. Dr. Zavlin reviewed and revised the manuscript.

https://orcid.org/0000-0003-3753-5511
