# Supplementary material for: The Burden of Physicians’ Administrative Workload Affects Clinical and Scientific Mentoring
Source: MedEdPublish (2016). 2019 Apr 1;8:74. [Version 1] doi: 10.15694/mep.2019.000074.1 (PMC10712589; doi:10.15694/mep.2019.000074.1)
Supplement: Peer review history

# Reviewer report: Reviewer response for version 1

**DOI:** 10.21956/mep.19828.r29874

**Published:** 2019-07-30

**Authors:** Jayne Garner

**Affiliations:** 1 university of liverpool

This review has been migrated. The reviewer awarded 4 stars out of 5 Thank you for raising these issues around mentoring and workload - relevant in the US and internationally. Providing evidence on the benefits of protected time for mentoring, teaching, feedback and CPD would be timely and relevant.

Reviewer Expertise:

NA

No decision status is available

# Reviewer report: Reviewer response for version 1

**DOI:** 10.21956/mep.19828.r29878

**Published:** 2019-04-26

**Authors:** Mohamed Al-Eraky

**Affiliations:** 1 University of Dammam

This review has been migrated. The reviewer awarded 3 stars out of 5 Thank you for this interpreting perspective. I 'partially agree' with the authors that face-to-face meetings are important in mentoring relationships. Yet, it’s not about the durations and frequency of these meetings, but the quality of mentoring. Mentoring is a skill that can be taught and assessed. That’s why we need to acknowledge mentoring as a standalone role, among others, played by physicians or faculty members, such as: patient care, teaching duties and administrative work. One solution can be offering options for practitioners to excel in one or two of the above roles, not all of them. This will create different pathways for their professional development, depending on their interest and abilities. Kind regards, Mohamed Al-Eraky

Reviewer Expertise:

NA

No decision status is available

# Reviewer report: Reviewer response for version 1

**DOI:** 10.21956/mep.19828.r29879

**Published:** 2019-04-16

**Authors:** Trevor Gibbs

**Affiliations:** 1 AMEE

This review has been migrated. The reviewer awarded 4 stars out of 5 I cannot imagine that any faculty member reading this letter would not sympathise and agree with the author. I would fully support my co-reviewer in suggesting that we need more research into this area before we are past the point of no return

Reviewer Expertise:

NA

No decision status is available

# Reviewer report: Reviewer response for version 1

**DOI:** 10.21956/mep.19828.r29877

**Published:** 2019-04-09

**Authors:** Gert Van Zyl

**Affiliations:** 1 University of the Free State

This review has been migrated. The reviewer awarded 4 stars out of 5 What a well written letter that addresses two very important issues: administrative workload and academic mentorship. It goes without saying the impact of these wo issues on the academic environment. We all spend time on the first area of administration with significant impact on academic mentorship and other areas of research. An academic leadership challenge to be addressed! Please take your letter to the next level and do some reserach on the impact and provide some solutions to the problem. It will be a winner!

Reviewer Expertise:

NA

No decision status is available

# Reviewer report: Reviewer response for version 1

**DOI:** 10.21956/mep.19828.r29876

**Published:** 2019-04-05

**Authors:** Sateesh Babu Arja

**Affiliations:** 1 Avalon University School of Medicine

This review has been migrated. The reviewer awarded 4 stars out of 5 Very simple and well-written letter. It is very interesting to see this letter from an MD-Ph.D student. Mentee and mentor relationship is very important to provide feedback regarding assessments and research work. This feedback is essential to improve learning. This feedback should be provided in a timely manner and be specific to be effective. This requires a lot of faculty's time and faculty development activities. I can understand the concern of the author of non-availability of time to the faculty due to physicians' administrative workload. This is a problem yet to find a solution.

Reviewer Expertise:

NA

No decision status is available

# Reviewer report: Reviewer response for version 1

**DOI:** 10.21956/mep.19828.r29875

**Published:** 2019-04-03

**Authors:** Johannes Hempe

**Affiliations:** 1 University of Kentucky

This review has been migrated. The reviewer awarded 5 stars out of 5 I read the article with interest and concur with many of the statements. From personal experience, I have seen many faculty spend much of their time on paperwork - time that could be used otherwise.

Reviewer Expertise:

NA

No decision status is available
